# Integrative SMR prioritizes oxidative stress–related regulatory genes for Alzheimer’s disease with brain-tissue validation

**DOI:** 10.1016/j.tjpad.2026.100535

**Published:** 2026-03-17

**Authors:** Liu Wu, Yu-Ting Dong, Xin Mu, Xiao Luo, Ze-Jun Chen

**Affiliations:** a Chengdu Integrated TCM & Western Medicine Hospital, Chengdu University of Traditional Chinese Medicine, Chengdu, Sichuan, China; b School of Acupuncture and Tuina, Chengdu University of Traditional Chinese Medicine, Chengdu, Sichuan, China; c Department of Neurology, Chengdu Integrated TCM& Western Medicine Hospital, Chengdu University of Traditional Chinese Medicine, Chengdu, Sichuan, China; d Department of Nephrology, Chengdu Integrated TCM& Western Medicine Hospital, Chengdu University of Traditional Chinese Medicine, Chengdu, Sichuan, China

**Keywords:** Alzheimer’s disease, oxidative stress, SMR, eQTL, mQTL, Enrichment analysis, Protein-protein interaction network

## Abstract

Oxidative stress (OS) plays a critical role in the pathogenesis of Alzheimer’s disease (AD), yet its genetic and epigenetic regulatory mechanisms remain unclear. In this study, we applied a three-step summary-based Mendelian randomization (SMR) framework to integrate Alzheimer’s disease (AD) GWAS summary statistics with peripheral-blood eQTL and mQTL datasets, and further evaluated brain-tissue relevance using GTEx v8 and AMP-AD resources. Across the three-step SMR analyses, we prioritized multiple OS-related candidate genes (e.g., CRLS1, PRKAA1, CYP2E1, GPX1, and APP) associated with AD risk, and brain-tissue analyses further highlighted KEAP1, SIRT1, and PRDX5 as region-relevant signals. Functional enrichment analyses highlighted critical pathways such as "Nrf2-mediated antioxidant response" and "PI3K-AKT signaling," emphasizing the roles of oxidative stress, mitochondrial function, and neuroinflammation in AD. Novel regulatory mechanisms were uncovered at methylation sites (e.g., cg20211653 associated with ABCA1), linking epigenetic regulation to transcriptional mechanisms and providing candidates for brain-tissue follow-up. This study provides new insights into the molecular underpinnings of AD, bridging genetic variation, epigenetic regulation, and transcription, and identifies potential therapeutic targets for mitigating oxidative damage and neurodegeneration.

## 1 Background

Alzheimer's disease (AD) is a progressive neurodegenerative disorder and the most common cause of dementia, affecting millions of people worldwide [1]. Oxidative stress (OS) is characterized by an imbalance between reactive oxygen species (ROS) production and the body's antioxidant defenses. Numerous studies have demonstrated that OS plays a pivotal role in the progression of AD by contributing to neuronal damage and dysfunction [2,3]. The brain is particularly susceptible to OS due to its high oxygen consumption and lipid-rich environment, making it vulnerable to lipid peroxidation and subsequent cell damage [4]. The oxidative damage further exacerbates key pathological processes of AD, such as the aggregation of amyloid-beta (Aβ) plaques and tau tangles, which are hallmarks of AD [5].

OS can lead to progressive loss of neurons and promote the occurrence and development of AD and various neurodegenerative diseases [6,7]. The brain’s high metabolic rate and limited regenerative capacity make it particularly vulnerable to ROS accumulation, which accelerates neurodegeneration and cognitive decline [8]. In addition to neurodegeneration, OS has been linked to other chronic conditions, including cardiovascular diseases, diabetes, and cancer, further highlighting its systemic role in disease pathogenesis [9,10]. The growing body of evidence underscores the importance of understanding the molecular mechanisms of OS in these diseases, particularly in AD, where it may serve as a potential therapeutic target [[11], [12], [13]]. However, the precise genetic mechanisms by which OS drives AD remain largely unexplored. By focusing on OS-related genes and their eQTL, we aim to identify novel genetic loci that may play a role in AD development.

Summary-based Mendelian Randomization (SMR) is a method that integrates GWAS and expression quantitative trait loci (eQTL) data to infer causal relationships between gene expression and disease [14]. By linking gene expression levels to genetic variants associated with disease, SMR helps to overcome the limitation of GWAS by offering a mechanistic interpretation of how genetic variants influence disease risk [15]. Furthermore, the Heterogeneity Estimation and Diagnostics in Meta-analysis (HEIDI) test within SMR assesses whether the observed association is due to a shared causal variant, providing a robust framework for validating colocalization signals [16]. Another key advantage of the HEIDI test is reducing confounding effects caused by linkage disequilibrium, and ensuring the directly related signals [17]. In addition to SMR and colocalization, functional enrichment analysis such as Gene Ontology (GO), Kyoto Encyclopedia of Genes and Genomes(KEGG), and Reactome pathways is able to further interpret the biological significance of the identified genes [18]. These tools allow researchers to summarize the genes into meaningful biological processes and pathways, shedding light on the underlying mechanisms driving disease pathology [19]. Through protein-protein interaction (PPI) network construction and functional module analysis, we intend to highlight key genes that could serve as potential therapeutic targets for AD.

The integration of eQTL and mQTL data with GWAS results provides a powerful framework to uncover regulatory mechanisms linking genetic variants to gene expression and epigenetic modifications. Moreover, brain-specific datasets, such as those from GTEx and AMP-AD, offer an unprecedented opportunity to study the molecular underpinnings of AD in critical brain regions, including the hippocampus and frontal cortex. In this study, we extended the traditional three-step SMR approach by integrating AD GWAS summary statistics with peripheral-blood eQTL and mQTL datasets, and subsequently assessing brain-tissue relevance using GTEx v8 and AMP-AD resources to characterize the regulatory landscape of OS–related genes in AD. The integration of blood-based eQTL and mQTL data with GWAS results provides a powerful framework to uncover regulatory mechanisms linking genetic variants to transcription and DNA methylation. In addition, brain-tissue resources (e.g., GTEx and AMP-AD) enable evaluation of tissue relevance for prioritized signals in AD-related regions. In this study, we performed a three-step SMR analysis primarily using peripheral-blood eQTL and mQTL datasets, and then assessed brain-tissue relevance using GTEx v8 and AMP-AD.

**Fig. 1:**
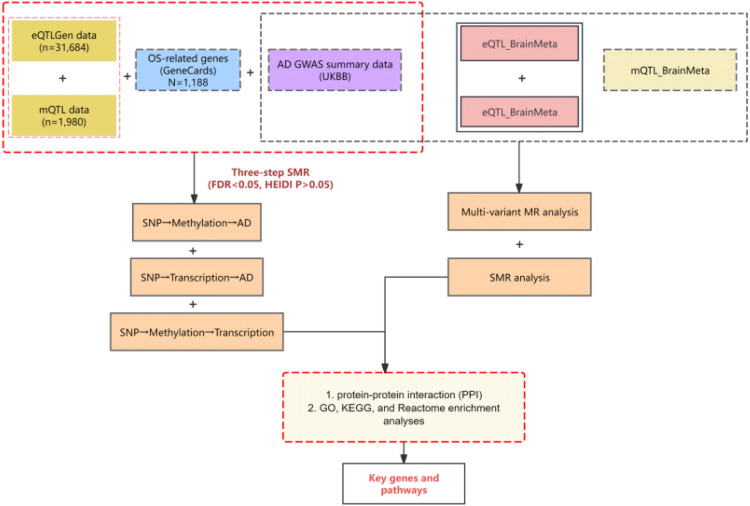
Description of the Workflow.

**In this study, we hypothesize that specific OS-related genetic variants are causally linked to AD through their regulation of gene expression.** Using SMR, we will integrate data from GWAS and eQTL/mQTL studies to test this hypothesis and identify colocalized loci where both disease risk and gene expression are driven by the same underlying variant. Furthermore, we hypothesize that these loci are enriched in biological pathways related to oxidative stress, neuroinflammation, and cell death, which are critical processes in AD pathology. By conducting GO, KEGG, and Reactome enrichment analyses, we aim to map the functional landscape of these loci and determine their broader biological relevance. By combining multi-omics data through SMR and functional enrichment analysis, this study aims to investigate the causal relationship between OS-related genes and AD, to provide a more comprehensive understanding of the genetic regulation in AD and to identify potential therapeutic targets, potentially paving the way for novel treatment strategies (Fig. 1 **is the flow diagram**).

## 2 Methods

### 2.1 Data sources

**GWAS Summary Data:** Genetic association statistics for AD liability were obtained from UK Biobank resources hosted by the Neale Lab. The phenotype is based on an AD-related polygenic risk score (PRS)–derived liability metric in 361,194 participants **(details in Suppl Table 1.1)**. We acknowledge that PRS-derived phenotypes are not equivalent to conventional case–control AD GWAS, and interpret SMR results accordingly.

**eQTL Data:** The eQTL data was from the eQTLGen Consortium, a resource integrating 37 datasets of 31,684 individuals. The data was generated from various tissue types and included comprehensive expression quantitative trait loci (eQTL) associations. The data is accessible through the consortium’s public database (https://www.eqtlgen.org/) and provides high-quality, large-scale eQTL information suitable for downstream analysis, including integrative analyses with GWAS data [20].

**mQTL Data:** Methylation quantitative trait loci (mQTL) data was sourced from a meta-analysis of two peripheral blood cohorts: the Brisbane Systems Genetics Study (BSGS) and the Lothian Birth Cohorts (LBC) [21,22].

**OS-Related Genes:** OS-related genes were retrieved from the GeneCards database, using a scoring system to prioritize genes with the highest relevance to OS [23].

**GTEx v8 Database:** Tissue-specific eQTL data for brain regions, including the hippocampus, frontal cortex, and substantia nigra (https://gtexportal.org/). These data allowed for brain-specific gene expression validation.

**AMP-AD Knowledge Portal:** Gene expression and methylation data from brain tissue samples of AD patients (https://adknowledgeportal.synapse.org/). This dataset was used for external validation of SMR results.

**STRING Database v11.5:** Protein-protein interaction (PPI) data for network construction and hub gene identification (https://string-db.org/).

### 2.2 SMR and Heidi analysis

The SMR analysis was adopted to explore the causal relationships between OS-related genes, transcription levels, and risk of AD. The SMR method allows for the integration of GWAS data with both eQTL and mQTL data, enabling the identification of pleiotropic effects that may underlie AD risk. SMR Tool: We used the SMR software v1.03 (https://yanglab.westlake.edu.cn/software/smr/) for all analyses [14]. Parameters: SMR analysis was conducted with a false discovery rate (FDR) of 〈 0.05 and HEIDI test p 〉 0.05 to filter out signals that were likely to be driven by linkage disequilibrium rather than pleiotropy [14]. Data Integration: The GWAS summary data were integrated with both eQTL and mQTL data to investigate the effect of SNPs on gene expression and methylation, and their association with AD.

### 2.3 PPI network analysis

To investigate the functional relationships among the identified genes, we constructed a PPI network using STRING v11.0 [24]. Criteria: Genes from the SMR analysis were used as input, and interaction scores were set at a confidence threshold of 0.7. Network Visualization: The network was visualized using Cytoscape v3.8.2, and functional modules were identified using the Molecular Complex Detection (MCODE).

### 2.4 Functional enrichment analysis

We conducted GO, KEGG, and Reactome pathway enrichment analyses to identify overrepresented biological processes and pathways among the genes identified by SMR analysis. Tools: The enrichment analysis was performed using DAVID v6.8 and ClusterProfiler [25,26]. Gene Sets: Genes significantly associated with AD risk (FDR < 0.05 in SMR) were used as input for the enrichment analysis. Thresholds: A p-value threshold of < 0.05 and an FDR-adjusted p-value of < 0.05 were used to identify significantly enriched terms. To conclude the results section, we have conducted a similarity analysis between the three steps of SMR based on GO, KEGG, and Reactome enrichment results. The analysis uses Jaccard similarity to assess the overlap in enriched pathways across the three steps.

## 3 Results

### 3.1 Three-step SMR analysis results

Using SMR based on abstract data, we identified several genes that are significantly associated with AD risk by transcription level, mainly in the following three steps: 1. SNP→Transcription→AD, 2. SNP→Methylation→AD, 3. SNP→Methylation→Transcription. These genes met the predefined threshold of FDR < 0.05 and p_HEIDI > 0.05, indicating that the SNPs affecting the transcription of these genes are likely to be causally related to AD, that the associations are consistent with a shared causal variant and show no strong evidence of heterogeneity (HEIDI *p* > 0.05).

In the SNP→Transcription→AD analysis using SMR, we identified several genes significantly associated with AD risk through their transcription levels (FDR 〈 0.05, p_HEIDI 〉 0.05) (The results are shown in Table 1). Among these, CRLS1 (FDR = 0.0209, p_HEIDI = 0.051) and PRKAA1 (FDR = 0.0409, p_HEIDI = 0.083) emerged as key candidates, highlighting their potential roles in AD pathogenesis.

**Table 1:** SNP→Transcription→Positive results of AD smr (FDR<0.05 and p_HEIDI > 0.05).

geneSYMBOL	topSNP	b_SMR	se_SMR	FDR	p_HEIDI
CYP2E1	rs1870726	−0.00051	0.00016	1.93E-03	9.98E-01
MSRA	rs6987305	0.00031	0.000101	2.12E-03	8.84E-01
HADHB	rs7603343	0.00082	0.00032	9.86E-03	7.91E-01
NUBPL	rs8003903	0.00036	0.00014	1.00E-02	9.81E-01
NR3C2	rs13104796	0.00151	0.000614	1.34E-02	6.86E-01
PPP1R15A	rs6053130	0.00092	0.00037	1.34E-02	1.61E-01
HSPA4	rs72801474	−0.000341	0.00014	1.43E-02	7.93E-01
FXN	rs564	−0.00128	0.00052	1.46E-02	8.13E-01
EIF2S1	rs41313505	0.00043	0.000179	1.49E-02	3.08E-01
BCL2L11	rs113135335	−0.0010	0.000439	1.86E-02	9.41E-01
STAT3	rs4305	−0.00172	0.00074	2.05E-02	4.81E-01
CRLS1	rs6017938	−0.00151	0.00065	2.09E-02	5.10E-02
APP	rs9618717	−0.000348	0.000156	2.51E-02	9.60E-01
TRIT1	rs79540048	0.000264	0.000119	2.62E-02	7.02E-01
TIA1	rs7585058	0.000473	0.000219	3.12E-02	5.52E-01
ACP1	rs300753	0.000799	0.00038	3.16E-02	2.05E-01
GPX1	rs11130203	0.000901	0.00042	3.27E-02	1.96E-01
NDUFS4	rs4147744	−0.0005701	0.000268	3.34E-02	9.35E-01
PNPT1	rs782642	0.00059	0.00028	3.45E-02	7.34E-01
APEX1	rs11628818	0.00017	7.95E-05	3.61E-02	5.04E-01
PRKAA1	rs3805487	0.0011	0.000539	4.09E-02	8.30E-02
CYGB	rs2007647	−0.000699	0.000346	4.32E-02	3.80E-01
NDUFA2	rs778594	0.000261	0.00013	4.61E-02	8.86E-01
RPS6KA5	rs61759760	0.000389	0.0001973	4.85E-02	2.97E-01

**Fig. 2:**
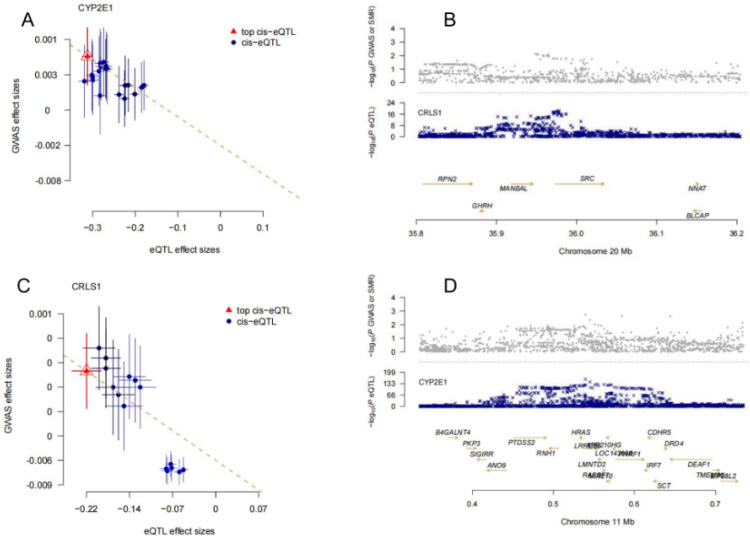
**Panel A and C**: This plot shows the correlation between SNP effects on the expression of the CRLS1 and CYP2E1 gene (eQTL) and their effects on AD risk (GWAS). The x-axis represents eQTL effect sizes, while the y-axis represents the effect sizes from GWAS analysis. Each point corresponds to a SNP, with the red triangle indicating the top cis-eQTL SNP, and the blue dots representing other SNPs associated with CRLS1 and CYP2E1 expression. **Panel B and D**: LocusZoom Plot for CRLS1 and CYP2E1. This LocusZoom plot displays the chromosomal region surrounding the CRLS1 and CYP2E1 gene, showing the significance of SNPs in both GWAS (top) and eQTL (bottom) analyses. The x-axis represents the chromosomal position, and the y-axes show the -log10(p-value) for SNPs from the GWAS (upper part) and eQTL (lower part) analyses. The significant overlap of SNPs with high -log10(p-values) in both GWAS and eQTL analyses provides evidence that these SNPs may regulate CRLS1 expression and also influence AD risk.

Notable findings also included GPX1, STAT3, and the well-known AD-related gene APP, whose transcriptional regulation aligns with the amyloid-beta hypothesis of AD. To further illustrate these associations, we selected two representative genes, CRLS1 and CYP2E1, for visualization (Fig. 2). Correlation plots depict the relationship between eQTL and GWAS effect sizes, while LocusZoom plots highlight significant SNPs in genomic regions linked to these genes. These findings underscore the critical role of transcriptional regulation in mediating AD risk.

Among the significant findings in the SNP→Methylation→AD analysis, several methylation sites demonstrated strong associations with AD risk. Notably, cg20211653 (ABCA1, FDR = 6.28E-03, p_HEIDI = 7.58E-01) and cg02272150 (CALM1, FDR = 7.14E-03, p_HEIDI = 8.46E-01) emerged as key loci. Other significant sites included cg01188578 (HADHA, FDR = 9.13E-03, p_HEIDI = 1.00) and two sites linked to ACE (cg04199256 and cg21657705, FDR = 9.30E-03 and 1.68E-02, respectively). These results suggest potential epigenetic regulation of genes involved in lipid metabolism and mitochondrial function. Additional findings highlighted methylation sites associated with VARS2, including cg15848685 and cg12457901, which showed consistent negative associations with AD risk (FDR = 1.05E-02 to 1.15E-02). Genes like PPARGC1A (FDR = 1.09E-02) and AHR (FDR = 1.11E-02) also stood out, emphasizing their roles in mitochondrial biogenesis, oxidative stress, and AD-related processes (Table 2 **presents the top 20 significant genes**).

**Table 2:** SNP→Methylation→AD: Top 20 SMR associations (FDR 〈 0.05; p_HEIDI 〉 0.05).

probeID	topSNP	b_SMR	se_SMR	FDR	p_HEIDI
cg20211653	rs55737841	−0.00028	0.000102877	6.28E-03	7.58E-01
cg02272150	rs2300502	0.000286	0.000106462	7.14E-03	8.46E-01
cg01188578	rs7603343	9.49E-05	3.64E-05	9.13E-03	1.00
cg04199256	rs4309	−0.00072	0.000278457	9.30E-03	9.69E-01
cg15848685	rs2596500	−0.000196	7.66E-05	1.05E-02	9.97E-01
cg19701879	rs4469064	0.000304	0.000119393	1.09E-02	9.54E-01
cg15978899	rs2596500	−0.00025	9.83E-05	1.11E-02	9.84E-01
cg12457901	rs2523593	−0.000186	7.30E-05	1.11E-02	9.99E-01
cg09249682	rs17347592	0.000523	0.000206015	1.11E-02	5.94E-01
cg10158679	rs2596500	−0.00026	0.000104208	1.12E-02	9.94E-01
cg14935711	rs2523593	−0.00020	8.03E-05	1.13E-02	9.97E-01
cg00933603	rs2596500	−0.00029	0.000112459	1.15E-02	9.70E-01
cg00244776	rs2596500	−0.000286	0.000112866	1.15E-02	9.89E-01
cg02149965	rs2596500	−0.00029	0.000115872	1.16E-02	9.81E-01
cg14581475	rs1888235	0.000178	7.09E-05	1.21E-02	8.65E-01
cg14184729	rs4983384	−0.0003168	0.000127251	1.28E-02	8.43E-01
cg17326313	rs11124572	−0.000691	0.000278403	1.30E-02	5.49E-01
cg26467571	rs2523593	−0.000463	0.000190765	1.52E-02	9.84E-01
cg21657705	rs4353	−0.000271	0.000113327	1.68E-02	6.26E-01
cg25620797	rs782579	0.0002329	9.95E-05	1.93E-02	5.77E-01

**Fig. 3:**
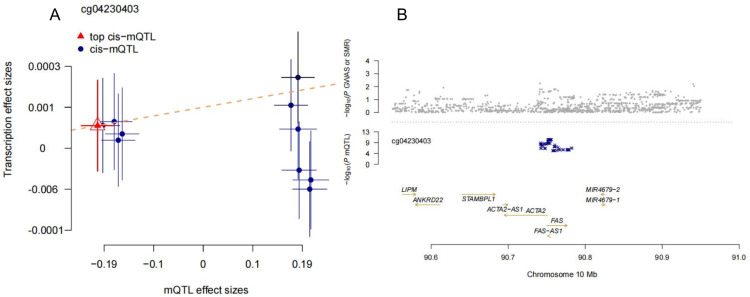
In the SNP → Methylation → Transcription analysis, we visualized a representative locus, cg024320403, from the third step of SMR analysis. This visualization consists of two panels: Panel A shows the correlation between mQTL effect sizes and transcription effect sizes, while Panel B is a LocusZoom plot that illustrates the chromosomal location of the significant SNPs affecting methylation and transcription at this locus.

In the SNP→Methylation→Transcription analysis, several genes demonstrated significant methylation-transcription associations linked to AD risk. Notably, PRDX5 (FDR = 2.67E-08, p_HEIDI = 0.973) and NOTCH1 (FDR = 3.81E-05, p_HEIDI = 0.951) emerged as key regulators, with PRDX5 strongly associated with OS response and NOTCH1 implicated in transcriptional control. Similarly, CAT (FDR = 8.95E-10, p_HEIDI = 0.938) was highlighted for its role in detoxifying reactive oxygen species. Other notable findings included PRKCD (FDR = 3.36E-05) and BDNF (FDR = 0.000381), both linked to methylation sites associated with neuroinflammation and neuronal plasticity. Additionally, FOXO1 and MAP3K5, with significant methylation-transcription associations, were found to regulate apoptosis and cellular stress responses. ESR1 (FDR = 1.66E-09) was also identified as a key gene influencing transcriptional activity via methylation (The results are shown in **Suppl Table 1.2**). To illustrate these findings, Fig. 3 highlights the representative locus cg024320403, showing the relationship between mQTL and transcription effects. These results reveal potential causal pathways linking methylation changes to transcriptional regulation, emphasizing the roles of OS, apoptosis, and neuroplasticity in AD pathology.

### 3.2 Functional enrichment analysis results

Functional enrichment analysis further revealed the biological significance of the significant genes in the three-step SMR analysis, which showed that these genes were mainly involved in key processes such as OS response, mitochondrial function and neuroinflammation.

In GO analysis, significant genes were enriched in "oxidative stress response" (GO:0006979) and "regulation of reactive oxide metabolism" (GO:2000377), indicating that these genes play a central role in the antioxidant defense mechanism. In addition, "cellular stress response" (GO:0070887) and "mitochondrial function regulation" (GO:0034599) were also highly significant, further supporting the key roles of OS and mitochondrial dysfunction in AD **(Suppl Figure 2.1 displays a combined bubble plot illustrating the enrichment results for all three steps**). KEGG analysis showed that significant genes were enriched in "PI3K-AKT signaling pathway" (hsa04151) and "Nrf2 regulating antioxidant stress pathway" (hsa05210), suggesting that these pathways may play an important role in the pathological process of AD by regulating OS response and cell survival. In addition, "lipid metabolism pathway" (hsa0006629) was also significantly enriched, suggesting a potential association between lipid metabolism disorders and AD **(Suppl Figure 2.2 displays a combined circle chart summarizing these results** (Lipid metabolism (p-value = 3.56e-04), Oxidative phosphorylation (p-value = 7.89e-04), Protein processing in endoplasmic reticulum (p-value = 1.56e-03)). Reactome analysis further highlighted the central role of OS and signaling, with significant pathways including "KEAP1-NFE2L2 antioxidant stress pathway" (R-HSA-9640462) and "Cellular response to chemical stress" (R-HSA-2559586). These findings suggest that OS-related genes may affect the development and progression of AD by activating antioxidant signaling to regulate neuronal survival and stress response (**The combined bubble plot of Reactome pathwasy enrichment results in Suppl Figure 2.3**).

**Fig. 4:**
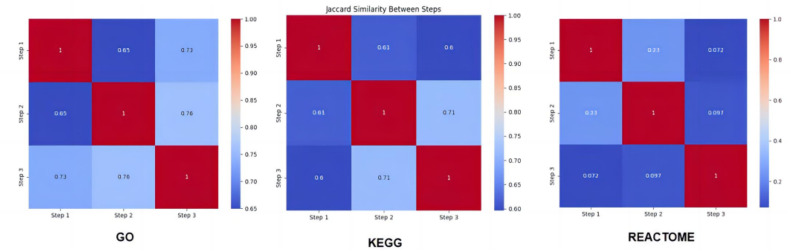
The heatmaps combines the results from GO, KEGG, and Reactome similarity analyses. The color gradient in each heatmap represents the Jaccard similarity coefficient, with red indicating higher similarity and blue representing lower similarity. Each matrix shows the pairwise similarity between the three SMR steps. GO and KEGG analyses reveal substantial pathway overlap between the steps, while Reactome shows lower overall similarity, emphasizing the different functional contexts captured by the Reactome database compared to GO and KEGG.

The GO enrichment analysis shows moderate overlap between the three steps, with Jaccard similarity scores ranging from 0.65 to 0.73, indicating a fair degree of commonality in the enriched pathways. KEGG enrichment also shows consistent similarity across the steps, with scores between 0.60 and 0.71, suggesting shared biological pathways between the different analyses. However, Reactome analysis displays a relatively lower similarity, with values as low as 0.072 between steps 1 and 3, highlighting more divergence in pathway enrichment in Reactome compared to GO and KEGG (Fig. 4).

### 3.3 Brain-tissue relevance assessment using GTEx and AMP-AD

To evaluate the brain-tissue relevance of the regulatory signals prioritized in the primary SMR analyses, we integrated AD GWAS summary statistics with brain-focused transcriptomic and epigenomic resources. Specifically, we leveraged GTEx (v8) brain-region eQTL data and AMP-AD brain tissue multi-omics data, focusing on key regions implicated in AD pathology, including the hippocampus, frontal cortex, and substantia nigra. This brain-tissue assessment aimed to determine whether prioritized loci showed consistent regulatory patterns in disease-relevant brain regions and to highlight plausible tissue-contextual regulatory mechanisms.

**Fig. 5:**
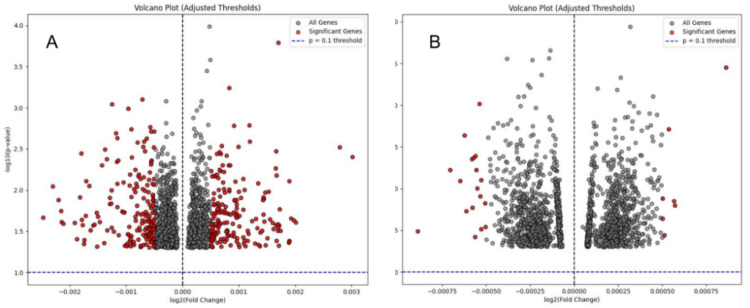
A: eQTL-SMR Volcano Plot for Brain Tissue. B: mQTL-SMR Volcano Plot for Brain Tissue. The x-axis represents the log2 fold change of SNP effects on gene expression, while the y-axis shows the -log10(p-value) for the association between SNPs and AD risk. Significant genes (FDR 〈 0.05, p_HEIDI 〉 0.05) are highlighted in red.

By combining GWAS with mQTL and eQTL data, SMR analysis identified 35 significant genes (FDR 〈 0.05, p_HEIDI 〉 0.05) across multiple brain regions (Fig. 5 **shows the visualized volcano plot)**. Among these, several OS-related genes, including KEAP1, SIRT1, and CRLS1, emerged as key candidates linked to AD risk through transcriptional and epigenetic regulation. KEAP1: Identified in the hippocampus, the top SNP (rs4930698) demonstrated strong association with AD (FDR = 2.67E-08).

**Fig. 6:**
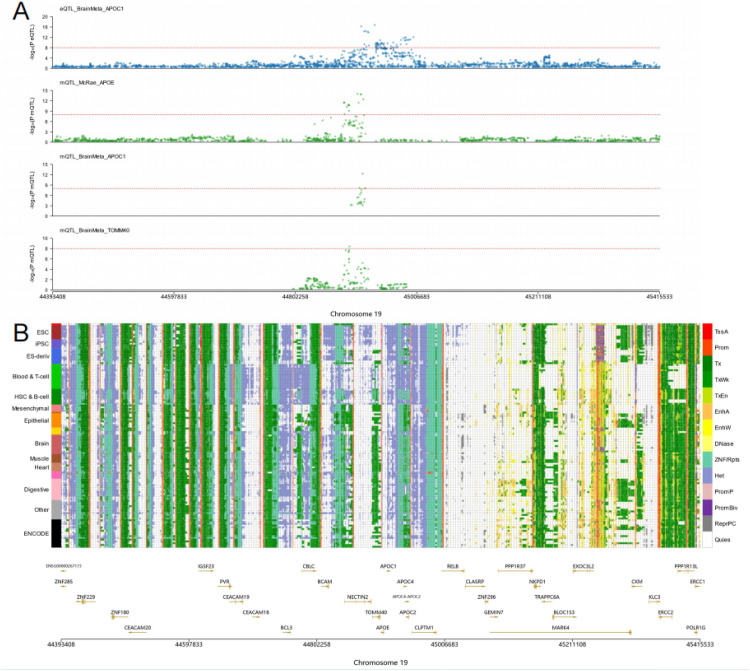
A. eQTL and mQTL signals in Chromosome 19. The top panel displays eQTL signals for APOC1 from BrainMeta, showing significant associations (*p* < 0.05). The remaining panels show mQTL signals for APOE, APOC1, and TOMM40, derived from BrainMeta and McRae studies, highlighting strong methylation-transcription linkages. **B.** Chromosome 19 functional annotation. The figure provides an overview of histone modifications, promoter activity, and regulatory elements across different tissues, based on data from ENCODE and Roadmap Epigenomics. Tissue types include brain, muscle, heart, digestive, epithelial, and immune cells. Genes such as APOE, APOC1, and TOMM40 are prominently featured, aligning with eQTL and mQTL findings.

KEAP1 is a central regulator of the Nrf2 antioxidant pathway, which protects neurons from oxidative damage. SIRT1: Significant associations were observed in both the frontal cortex (FDR = 1.89E-04) and substantia nigra (FDR = 2.13E-03), highlighting its role in mitochondrial biogenesis and neuroprotection. CRLS1: This mitochondrial gene showed consistent significance across multiple brain regions, including the hippocampus (FDR = 1.10E-03), supporting its involvement in maintaining mitochondrial membrane integrity and neuronal survival. In addition to these key genes, other notable findings include PRDX5 (FDR = 3.56E-05), involved in ROS detoxification, and TNFRSF1A (FDR = 2.91E-03), a mediator of neuroinflammation (Fig. 6).

Functional enrichment analysis with Brain revealed that the significant genes identified through SMR are involved in critical biological processes and pathways:GO Analysis: "Response to oxidative stress" (GO:0006,979, *p* = 3.21E-04) and "Mitochondrial function" (GO:0034,599, *p* = 2.85E-03) were among the most enriched terms, emphasizing the role of OS and mitochondrial dysfunction in AD pathology. KEGG Pathways: Key pathways included "Nrf2-mediated antioxidant response" (hsa05210, *p* = 1.12E-03) and "PI3K-AKT signaling pathway" (hsa04151, *p* = 2.31E-04). These pathways highlight mechanisms of neuronal survival and OS regulation. Reactome Pathways: Significant enrichment was observed in "KEAP1-NFE2L2 antioxidant response" (R-HSA-9640,462, *p* = 1.56E-03) and "Regulation of beta-cell development" (R-HSA-9707,603, *p* = 3.25E-04), suggesting potential links between metabolic regulation and AD risk.

Brain-specific mQTL/eQTL analysis revealed differential expression of key genes across brain regions: CRLS1 and SIRT1 showed significantly higher expression in the hippocampus and frontal cortex, regions heavily affected by AD pathology. These findings provide additional support for the tissue-specific regulatory roles of these genes in AD development.

### 3.4 PPI network

**In Suppl Figure 2.4**, the PPI network consists of 68 nodes and 59 edges, representing the proteins encoded by genes identified through the three-step SMR analysis and their known interactions. The red-colored nodes highlight the hub genes with the highest degree of connectivity, while the pink nodes represent other genes involved in the network. The connections (edges) between the nodes indicate known protein-protein interactions based on existing databases. The hub genes with the highest connectivity include KEAP1, SIRT1, TNFRSF1A, MAPK1, and NFKBIA, which have a central role in this network, connecting to several other proteins. These genes are prominent in their interaction patterns and may serve as key nodes in this biological system. Other highly connected genes include GSR, MAPK14, IL8, and NFKB1, each of which also shows substantial interaction with other proteins in the network. Key hub genes with their corresponding degrees of connectivity: KEAP1 (degree = 11), SIRT1 (degree = 9), TNFRSF1A (degree = 8), MAPK1 (degree = 8), NFKBIA (degree = 7). The PPI network highlights the interactions between these proteins and their relationship with the other genes involved, as displayed by the connecting edges. The layout of the network indicates a highly interconnected structure, with hub genes serving as central connectors between multiple nodes.

## 4 Discussion

This study utilized a three-step SMR analysis to investigate the genetic regulation of OS-related pathways in AD. By integrating AD GWAS summary statistics with peripheral-blood eQTL and mQTL datasetsusing a three-step SMR framework, and subsequently evaluating brain-tissue relevance using GTEx v8 and AMP-AD resources, we identified significant genes and pathways that shed light on the molecular mechanisms underlying AD pathology. The key findings of this study are as follows: Several OS-related genes, including KEAP1, SIRT1, and CRLS1, were identified as significantly associated with AD risk. KEAP1, a critical regulator of the Nrf2 antioxidant pathway, demonstrated strong associations with SNPs in the hippocampus (FDR = 2.67E-08). SIRT1, known for its neuroprotective effects, was identified in both the frontal cortex and substantia nigra. CRLS1, a gene essential for mitochondrial function, was consistently significant across multiple brain regions. The integration of brain-specific mQTL and eQTL data revealed differential expression of these genes across key brain regions implicated in AD pathology. For example, CRLS1 and SIRT1 showed significantly higher expression in the hippocampus and frontal cortex, regions known for their vulnerability to oxidative damage and neurodegeneration in AD. Functional enrichment analyses highlighted critical pathways associated with AD, including "Response to oxidative stress" (GO:0006979) and "Nrf2-mediated antioxidant response" (hsa05210). These findings underscore the central role of OS and mitochondrial dysfunction in the pathogenesis of AD. PPI network analysis identified KEAP1, SIRT1, and TNFRSF1A as hub genes with the highest degree of connectivity. These genes are integral to the antioxidant defense system, mitochondrial function, and neuroinflammatory response, respectively, suggesting their pivotal roles in AD-related molecular mechanisms. In summary, this study provides new insights into the genetic regulation of OS-related pathways in AD by combining multi-omics data from GWAS, mQTL, and eQTL analyses. The identification of tissue-specific regulatory genes and pathways not only enhances our understanding of AD pathogenesis but also highlights potential therapeutic targets for mitigating oxidative damage and neurodegeneration.

Our findings extend the mechanisms underlying the effects of OS on AD. Previous studies have highlighted the involvement of OS-related genes such as SIRT1 and KEAP1 in neurodegenerative processes, with SIRT1 being associated with neuroprotection and the modulation of AD pathology through its role in reducing oxidative damage and promoting mitochondrial function [27]. Similarly, KEAP1 has been identified as a key regulator of the OS response via the Nrf2 pathway, and its dysregulation is linked to increased susceptibility to oxidative damage in neurons [28]. Our identification of KEAP1 and SIRT1 as hub genes in the PPI network reinforces their central role in AD, consistent with these previous findings. Moreover, our study identifies novel associations, such as the involvement of CRLS1, PRKAA1, and GPX1 in the SNP-transcription-AD analysis. While GPX1 has been previously recognized for its role in mitigating OS by reducing hydrogen peroxide [29], its direct involvement in AD has been less explored. The presence of CRLS1, a gene involved in mitochondrial lipid metabolism, in our analysis highlights the critical role of mitochondrial dysfunction in AD, corroborating earlier studies that have proposed mitochondrial damage as a key player in neurodegeneration [30,31]. Similarly, the role of PRKAA1 (AMPKα1) in energy homeostasis and its regulation of neuronal survival under metabolic stress conditions is well-established [32,33]. However, its specific contribution to AD pathology remains under investigation, and our findings provide new evidence supporting its relevance.

One significant divergence from prior studies is the identification of methylation sites such as cg20211653 associated with ABCA1 and cg02272150 associated with CALM1 in the SNP-methylation-AD pathway. While methylation changes in AD have been studied, the specific involvement of these loci has not been extensively reported [34]. The involvement of ABCA1, a gene known for its role in cholesterol metabolism and amyloid-β clearance [35], highlights a potential epigenetic regulation mechanism that may influence AD risk. Similarly, CALMII (calmodulin 2), a gene involved in calcium signaling, has been linked to synaptic plasticity and neuronal function [36], suggesting that methylation changes may affect its expression in the context of AD. The identification of PRDX5 and CAT in the transcription-methylation-AD pathway offers new insights into antioxidant defense mechanisms in AD. While both PRDX5 and CAT have been associated with the detoxification of ROS [37,38], their direct involvement in AD through methylation changes is a novel finding. This suggests that epigenetic regulation of OS response genes could be a crucial factor in AD pathology, complementing existing research that primarily focuses on transcriptional changes.

The APOE–TOMM40 region is a well-established AD risk locus, and seminal work has highlighted APOE–TOMM40 allelic variation with metabolic implications relevant to AD susceptibility. The interplay between OS, mitochondrial dysfunction, and neuroinflammation emerges as a central theme in the pathogenesis of AD, aligning with well-established hypotheses regarding the disease’s underlying mechanisms. One potential mechanism involves the dysregulation of redox homeostasis driven by impaired antioxidant defense systems. Genes such as SIRT1, GPX1, PRDX5, and CAT, identified in our analysis, are key regulators of the cellular response to OS. SIRT1 has been shown to enhance mitochondrial biogenesis and antioxidant defenses by deacetylating transcription factors such as PGC-1α [39]. This mechanism directly impacts the regulation of mitochondrial function and the reduction of oxidative damage in neurons. Similarly, GPX1 and CAT are responsible for neutralizing ROS, particularly hydrogen peroxide, thereby mitigating oxidative damage to DNA, lipids, and proteins [40,41]. The dysregulation of these genes may contribute to the accumulation of oxidative damage, a hallmark of AD pathology [42]. The identification of KEAP1 as a hub gene suggests another key pathway related to OS response and we speculate that it may be mainly through the KEAP1-nrf2 signaling axis. Under conditions of OS, KEAP1-mediated inhibition of Nrf2 is alleviated, allowing Nrf2 to translocate to the nucleus and activate the transcription of antioxidant genes [43]. This protective mechanism can be compromised in AD, as evidenced by reduced Nrf2 activity in AD brains [44]. Consequently, the decreased ability to mount an effective antioxidant response exacerbates oxidative damage, contributing to neuronal loss.

The chromosome 19 APOE region is a well-established AD risk locus, and seminal work has highlighted APOE–TOMM40 allelic variation with important metabolic implications for AD susceptibility [45,46]. In this context, our convergent regulatory signals across APOE-region genes (APOE/APOC1/TOMM40) provide a plausible regulatory bridge from genetic architecture to epigenetic and transcriptional control. Mechanistically, coordinated regulation within this locus may perturb lipid handling and mitochondrial–metabolic stress responses, amplify oxidative stress signaling, and thereby accelerate downstream neurodegenerative cascades. These observations motivate locus-specific, brain-region and cell-type–resolved validation of APOE-region regulatory mechanisms.

Another significant mechanism may involve mitochondrial dysfunction, which has long been implicated in AD [8]. Genes such as CRLS1 and HADHB, both identified in our analysis, are critical for mitochondrial function. CRLS1 is involved in cardiolipin biosynthesis, which is essential for maintaining mitochondrial membrane integrity and function [47,48]. Deficiencies in cardiolipin can lead to mitochondrial dysfunction and the release of pro-apoptotic factors, further contributing to neuronal death in AD [49]. HADHB, a key player in fatty acid β-oxidation within the mitochondria, may also influence mitochondrial energy metabolism, linking metabolic stress to neurodegeneration [50]. Beyond mitochondrial dysfunction, the involvement of genes such as APP (amyloid precursor protein) and BCL2L11 (BIM) in our analysis suggests a convergence of OS and apoptosis pathways in AD. APP, through its cleavage products such as amyloid-β, has been implicated in the generation of ROS [51]. The accumulation of amyloid-β in the brain can exacerbate OS by impairing mitochondrial function and inducing lipid peroxidation [52]. Furthermore, BCL2L11, a pro-apoptotic member of the BCL-2 family, may mediate OS-induced apoptosis in neurons [53]. Elevated levels of OS can trigger the intrinsic apoptotic pathway via mitochondrial cytochrome c release, which is regulated by the balance between pro- and anti-apoptotic BCL-2 family members [54]. Methylation-related changes identified in genes such as ABCA1 and CALM1 provide further insight into the epigenetic regulation of AD-related pathways. ABCA1 plays a critical role in lipid transport and amyloid-β clearance [55,56], and its downregulation via epigenetic mechanisms could impair amyloid clearance, exacerbating AD progression. CALM1, involved in calcium signaling, may also be epigenetically regulated in AD, as dysregulated calcium homeostasis is a known contributor to synaptic dysfunction and neurodegeneration [57]. Aberrant methylation of these genes could lead to altered expression levels, contributing to AD pathology by modulating amyloid metabolism and neuronal excitability [58]. These observations can be interpreted within the “calcium hypothesis” framework, which proposes that disrupted neuronal Ca²⁺ homeostasis is a central mediator linking upstream stressors to synaptic failure and neurodegeneration [59,60]. Importantly, oxidative stress and Ca²⁺ dyshomeostasis form a feed-forward loop: ROS can impair Ca²⁺ handling and ER–mitochondria coupling, whereas Ca²⁺ overload exacerbates mitochondrial dysfunction, amplifies ROS production, and promotes intrinsic apoptotic signaling [60]. This OS–Ca²⁺–mitochondria axis has also been discussed as a plausible mechanism through which vascular–metabolic stressors and aging accelerate cognitive decline, including in mixed and post-stroke dementia contexts.

Furthermore, the involvement of transcriptional regulators such as STAT3 and NFKB1 (NF-κB) in our analysis points to the role of neuroinflammation in AD. STAT3 is a transcription factor involved in the inflammatory response, and its activation has been linked to neuroinflammation and AD [61]. Similarly, NF-κB, a master regulator of inflammation, is activated in response to OS and has been implicated in promoting neuroinflammation in AD [62]. The chronic activation of these inflammatory pathways, fueled by OS, likely contributes to the progressive neurodegeneration observed in AD [63]. Finally, our PPI network highlights interactions between key signaling molecules involved in OS and inflammatory responses, suggesting that these pathways do not operate in isolation but are part of an interconnected network driving AD pathogenesis. For example, interactions between TLR2 (Toll-like receptor 2) and MAPK1 (Mitogen-activated protein kinase 1) suggest a link between innate immune responses and OS pathways [[64], [65], [66]]. The crosstalk between OS, mitochondrial dysfunction, and neuroinflammation forms a complex network that may underlie the multifactorial nature of AD [67]. Our findings suggest that OS plays a pivotal role in AD by modulating mitochondrial function, apoptosis, and neuroinflammation. These results are consistent with and expand upon existing research, providing new insights into the molecular mechanisms linking OS to AD. Further exploration of these pathways may offer novel therapeutic targets for mitigating oxidative damage and neurodegeneration in AD [68,69].

While our study provides important insights into the role of OS genes in AD, several limitations should be noted. First, the use of GWAS summary statistics limits our ability to perform individual-level analyses, which may reduce the precision of the causal inferences. Second, the eQTL and mQTL data were derived from peripheral tissues, which may not fully reflect the gene regulatory processes occurring in the brain. Third, our analysis relied on available datasets, which might introduce bias due to population stratification and other confounding factors. Finally, although we identified key pathways, the study is largely correlative, and functional validation of these genes in AD pathophysiology is necessary to establish causality.

Building on the findings of this study, future research should prioritize functional validation of the identified OS-related genes and pathways in AD. Experimental approaches, such as CRISPR gene editing or RNA interference, could be employed to assess the specific roles of these genes in AD models. Additionally, integrating brain-specific multi-omics data, including single-cell transcriptomics and epigenomics, would provide a more precise understanding of gene regulation in relevant tissues. Longitudinal studies tracking gene expression and methylation changes over the course of disease progression could also shed light on the temporal dynamics of AD. Furthermore, exploring the interaction between OS genes and other environmental or genetic factors may provide new insights into personalized treatment strategies for AD.

## 5 Conclusion

This integrative SMR analyses prioritized OS–related regulatory signals for AD and mapped them to two interpretable mechanistic themes. The chromosome 19 APOE locus emerged as a regulatory hub with convergent eQTL/mQTL evidence across APOE-region genes (e.g., APOE/APOC1/TOMM40), supporting a testable model linking inherited regulation to lipid–mitochondrial biology and OS-responsive programs. In parallel, candidates such as ABCA1 and CALM1 suggest an OS–lipid–calcium–mitochondria axis through which epigenetic/transcriptional regulation may modulate calcium homeostasis, mitochondrial stress, and downstream neurodegenerative processes. These findings define concrete next steps for brain-region and cell-type–resolved fine-mapping/colocalization and functional validation to determine how prioritized regulatory mechanisms influence OS responses and neurodegeneration in AD models.

## Funding sources

This work was supported by the Sichuan Provincial Administration of Traditional Chinese Medicine (No. 25MSZX201) and the Chengdu Municipal Health Commission–10.13039/501100013792Chengdu University of Traditional Chinese Medicine Joint Project (No. WXLH202406002).

## Ethics statement

The data in this study are from public databases and do not require ethics committee approval.

## Data availability

The data used in this study were obtained from publicly available sources. Genetic data for AD were derived from the UK Biobank, available from the Neale Lab (http://www.nealelab.is/uk-biobank). Eqtl data were obtained from the eQTLGen Consortium (https://www.eqtlgen.org), mQTL data was sourced from a meta-analysis of two peripheral blood cohorts: BSGS and LBC, OS-related genes were retrieved from the GeneCards database. GTEx v8 Database: Tissue-specific eQTL data for brain regions, including the hippocampus, frontal cortex, and substantia nigra (https://gtexportal.org). AMP-AD Knowledge Portal: Gene expression and methylation data from brain tissue samples of AD patients (https://adknowledgeportal.synapse.org).

## Declaration of the use of generative AI and AI-assisted technologies in scientific writing and in figures, images and artwork

During the preparation of this work the author(s) used ChatGPT (OpenAI) in order to improve language clarity and readability (e.g., grammar and stylistic refinement). After using this tool/service, the author(s) reviewed and edited the content as needed and take(s) full responsibility for the content of the published article.

## CRediT authorship contribution statement

**Liu Wu:** Writing – review & editing, Writing – original draft, Visualization, Project administration, Methodology, Funding acquisition, Formal analysis, Conceptualization. **Yu-Ting Dong:** Writing – original draft, Software, Resources, Investigation, Data curation. **Xin Mu:** Writing – review & editing, Supervision, Resources, Project administration. **Xiao Luo:** Writing – review & editing, Validation, Resources, Investigation. **Ze-Jun Chen:** Writing – review & editing, Visualization, Validation, Methodology, Funding acquisition.

## Declaration of competing interest

The authors declare that they have no known competing financial interests or personal relationships that could have appeared to influence the work reported in this paper.

## Appendix Supplementary materials

Image, application 1

Image, application 2
