# Supplementary material for: Integrative SMR prioritizes oxidative stress–related regulatory genes for Alzheimer’s disease with brain-tissue validation
Source: J Prev Alzheimers Dis. 2026 Mar 17;13(5):100535. doi: 10.1016/j.tjpad.2026.100535 (PMC13014940; doi:10.1016/j.tjpad.2026.100535)
Supplement: Supplementary file 1 [file mmc1.docx]

**Suppl 1. Data sources and SNP → Methylation → Transcription**

**Suppl Table 1.1. Summary of Key Data Statistics**

**Suppl Table 1.2. SNP → Methylation → Transcription results of AD smr (p_SMR<0.05 and p_HEIDI > 0.05)**

| **Table 1. Summary of Key Data Statistics** | | | | |
| --- | --- | --- | --- | --- |
| **Dataset** | **Participants** | **Gene Count** | **SNP Count** | **P-value Threshold** |
| GWAS (AD) | 361,194 | - | >1 million | Derived from GWAS |
| eQTLGen (eQTL Gen) | 31,684 | >30,000 | >1 million | p < 5e-8 |
| mQTL (BSGS & LBC) | 1,980 | 450,000+ CpG | -- | p < 5e-8 |
| Brain_Meta eQTL | 2865 | - |  | p < 5e-8 |
| GTEx v8 eQTL(Brain) | 5439 | - | - |  |
| OS Genes (GeneCards) | - | 1,188 | - | Based on GeneCards score |
|  | | | | |

| **Table 1.2.** **SNP → Methylation → Transcription results of AD smr (p_SMR<0.05 and p_HEIDI > 0.05)** | | | | | |
| --- | --- | --- | --- | --- | --- |
| **Expo_ID** | **topSNP** | **b_SMR** | **se_SMR** | **p_SMR** | **p_HEIDI** |
| cg12453748 | rs4930698 | -1.59603 | 0.286963 | 2.67E-08 | 0.9730906 |
| cg13659914 | rs35116034 | 0.226098 | 0.0548915 | 3.81E-05 | 0.9506028 |
| cg02109652 | rs12793666 | -3.30724 | 0.539768 | 8.95E-10 | 0.9380582 |
| cg23168971 | rs9862206 | -0.263217 | 0.0634604 | 3.36E-05 | 0.931833 |
| cg03567652 | rs2738464 | -0.0296111 | 0.0136078 | 0.02955153 | 0.9191058 |
| cg06087101 | rs113038897 | -0.0230956 | 0.0113577 | 0.04200513 | 0.888273 |
| cg00298481 | rs11030107 | -0.277237 | 0.0780379 | 0.000381457 | 0.7923562 |
| cg03337430 | rs3990965 | 0.0959267 | 0.0440741 | 0.02951898 | 0.7917467 |
| cg23413567 | rs113377887 | -0.281829 | 0.0647319 | 1.34E-05 | 0.7747678 |
| cg16445842 | rs949037 | 0.105608 | 0.0356862 | 0.003082969 | 0.7691918 |
| cg07768201 | rs11604331 | -3.67581 | 0.674709 | 5.09E-08 | 0.7570388 |
| cg18219712 | rs3898649 | -0.0794293 | 0.0400598 | 0.04739346 | 0.7419445 |
| cg10599345 | rs3903660 | -1.86011 | 0.24428 | 2.64E-14 | 0.7341648 |
| cg22867714 | rs980130 | -0.0528092 | 0.0176237 | 0.002731139 | 0.7003825 |
| cg18940763 | rs2013784 | -1.65746 | 0.255989 | 9.50E-11 | 0.6960588 |
| cg24451117 | rs7743709 | -1.65426 | 0.190635 | 4.04E-18 | 0.6861409 |
| cg04355227 | rs7755197 | 0.236054 | 0.0647689 | 0.000267848 | 0.6579058 |
| cg11231349 | rs1415257 | -0.0550927 | 0.026341 | 0.03648183 | 0.6246172 |
| cg10381200 | rs891087 | -0.312748 | 0.0606193 | 2.48E-07 | 0.610257 |
| cg14225168 | rs3124599 | 0.0646535 | 0.0300838 | 0.03162534 | 0.6073356 |
| cg25027788 | rs7967155 | 0.0502997 | 0.0217423 | 0.02069798 | 0.6059126 |
| cg06611115 | rs851978 | -0.49536 | 0.0821704 | 1.66E-09 | 0.5871419 |
| cg25243385 | rs6511719 | 0.120426 | 0.0540049 | 0.02575298 | 0.5632488 |
| cg11353706 | rs2227674 | 0.340868 | 0.0639193 | 9.67E-08 | 0.5458163 |
| cg08418872 | rs4149576 | -0.123074 | 0.0457365 | 0.00712529 | 0.5365068 |
| cg16257983 | rs10887875 | -1.47176 | 0.192817 | 2.29E-14 | 0.5214957 |
| cg22567591 | rs2302432 | -0.293121 | 0.068598 | 1.93E-05 | 0.5032067 |
