# Supplementary material for: Integrative SMR prioritizes oxidative stress–related regulatory genes for Alzheimer’s disease with brain-tissue validation
Source: J Prev Alzheimers Dis. 2026 Mar 17;13(5):100535. doi: 10.1016/j.tjpad.2026.100535 (PMC13014940; doi:10.1016/j.tjpad.2026.100535)
Supplement: Supplementary file 2 [file mmc2.docx]

**Suppl 2. PPI network result and Functional Enrichment Analysis Results**

**Suppl Figure 2.1.** The combined bubble plots illustrating the GO enrichment results for all three SMR step.s


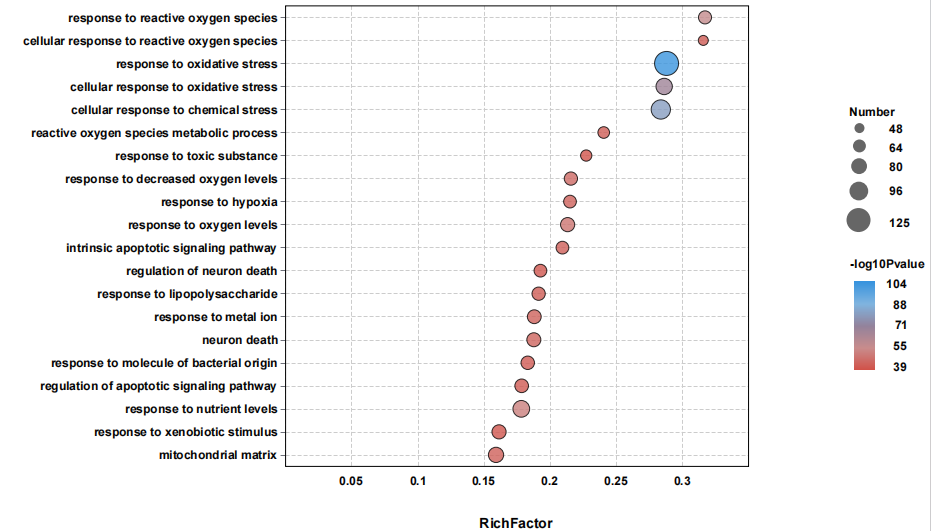


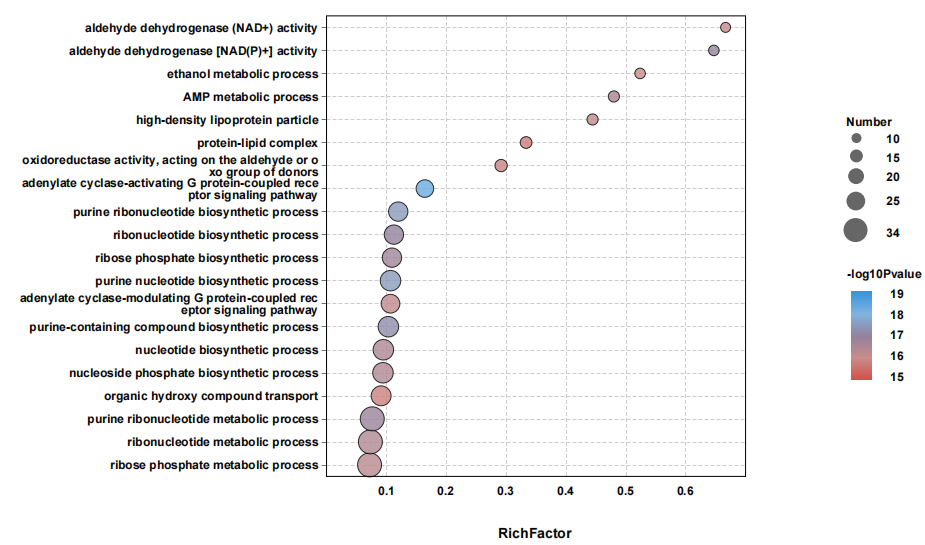


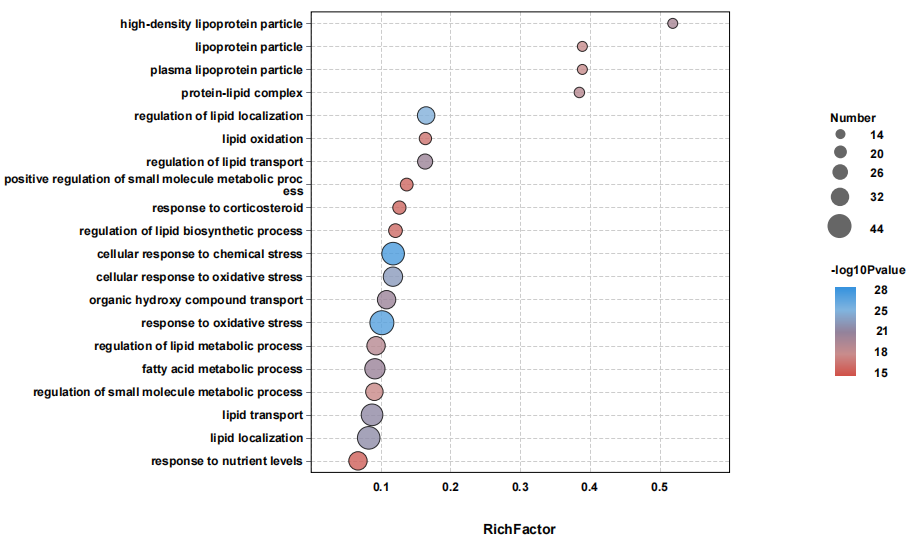
**SuSuppl Figure 2.1.** The chat shows a combined bubble plot of the GO enrichment results for all three steps of SMR analysis. The size of each bubble represents the number of genes associated with each GO term, while the color intensity reflects the significance level (-log10(p-value)) of the enrichment. The biological processes most significantly enriched across all three steps include OS response, metabolic processes, and regulation of reactive oxygen species, reinforcing the central role of oxidative damage in AD progression.

**Suppl Figure 2.2.** The combined circular plots illustrating the GO enrichment results for all three SMR steps.


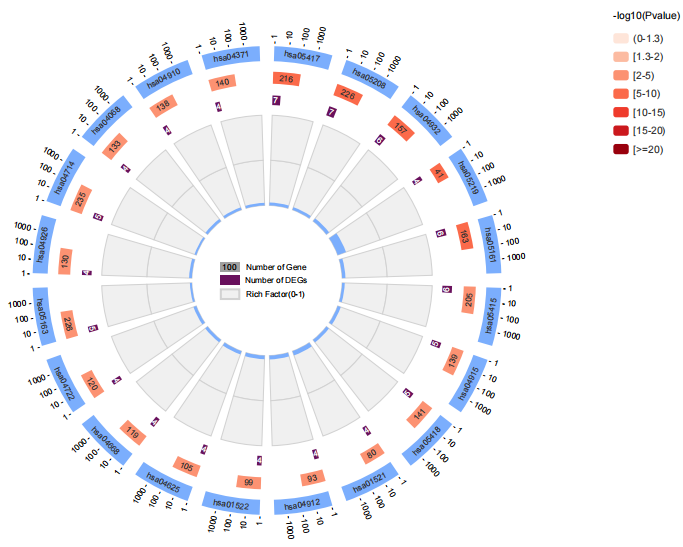


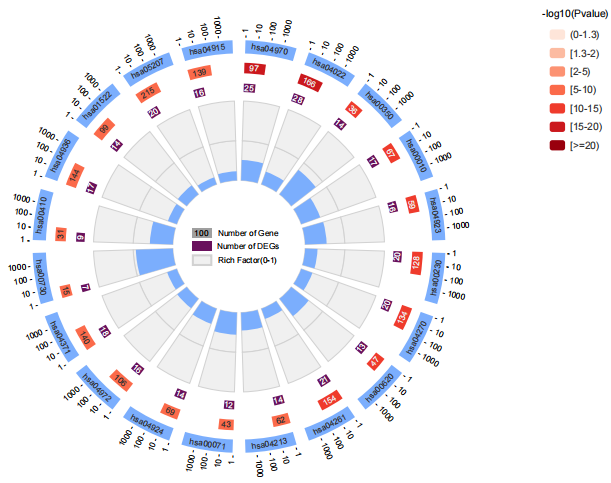


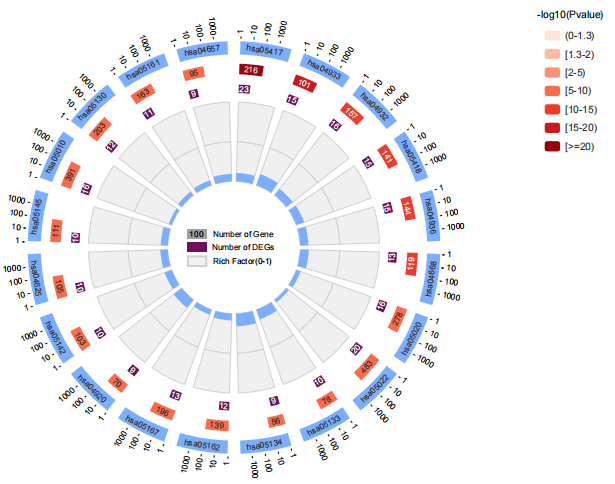


**Suppl Figure 2.2.** Presents the KEGG pathway enrichment results for the three-step SMR analysis using circular plots. Each circular plot represents the most significantly enriched pathways identified in each step: SNP → Transcription → AD, SNP → Methylation → AD, and SNP → Methylation → Transcription. The pathways are arranged around the circle, with the size of each segment corresponding to the number of genes involved in the pathway, while the color intensity reflects the significance level of the enrichment (-log10(p-value)). This visual representation allows for a clear comparison of the most important pathways across the three analyses, highlighting key metabolic, signaling, and OS-related pathways.

**Suppl Figure 2.3.** The combined bubble plots illustrating the GO enrichment results for all three SMR steps.


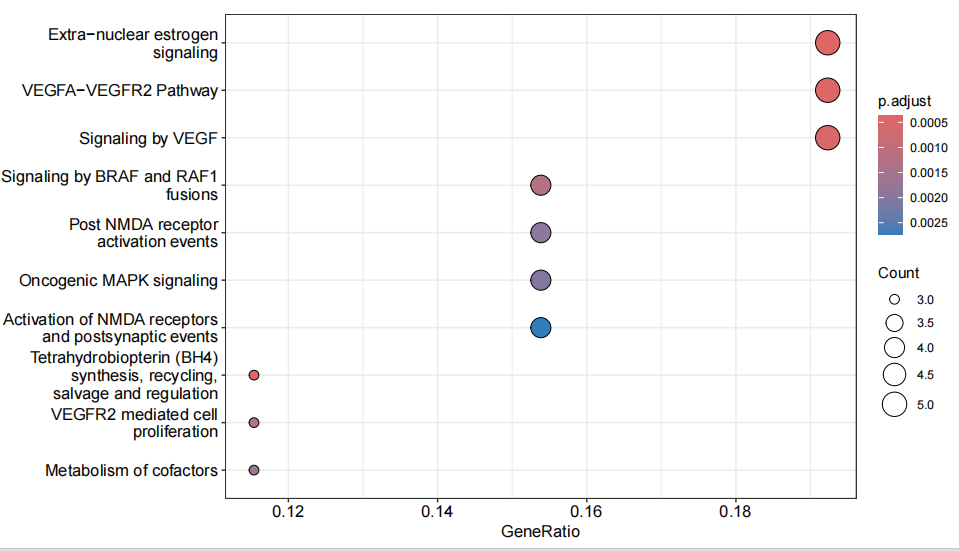


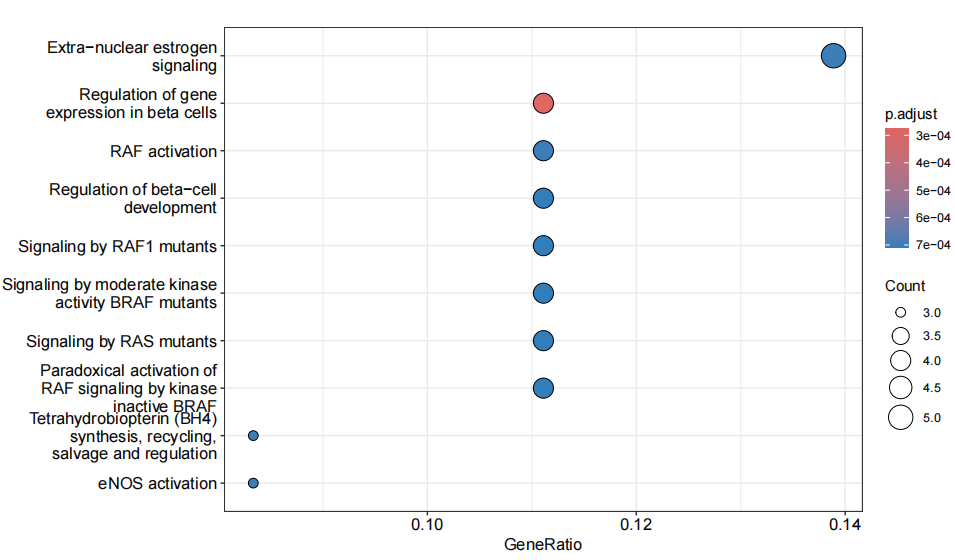


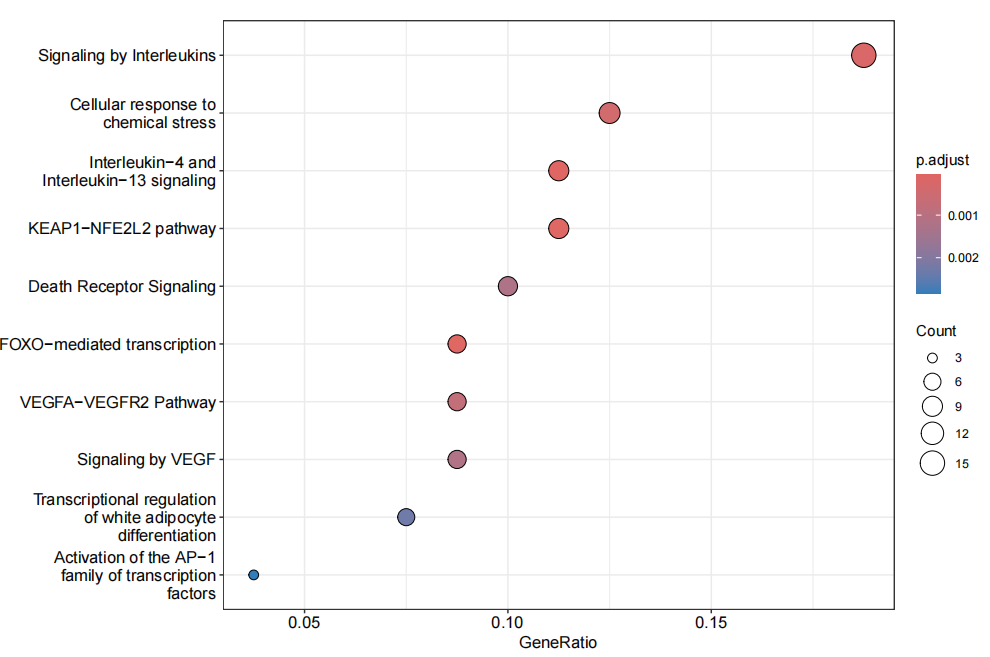


**Suppl Figure 2.3.** The chat shows the combined bubble plot for Reactome pathway enrichment across the three steps. Each bubble represents the number of genes involved in a specific pathway, with the size of the bubble proportional to gene count and the color intensity reflecting the significance level (-log10(p-value)).

**Suppl Figure 2.4.** PPI network result


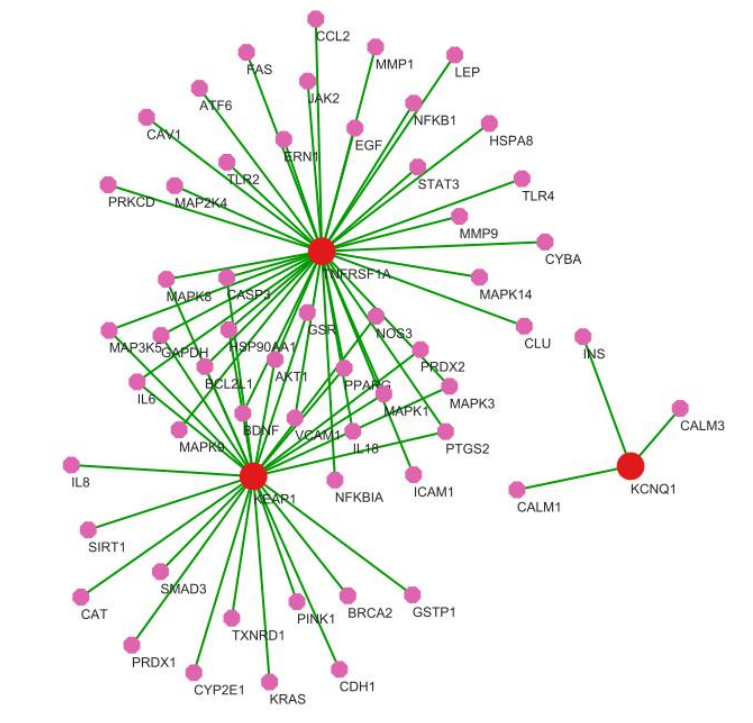


**Figure 2.4.** PPI network for OSs-related genes identified through three-step SMR analysis. The red nodes represent hub genes with high connectivity, while the pink nodes represent other genes in the network. The edges indicate known interactions between proteins. Hub genes include KEAP1, SIRT1, TNFRSF1A, MAPK1, and NFKBIA, which display the highest degree of connectivity.
